# Whole-genome sequence of an *Aspergillus parasiticus* strain isolated from Kenyan soil

**DOI:** 10.1128/MRA.00203-23

**Published:** 2023-07-18

**Authors:** Alexandra Schamann, Rolf Geisen, Markus Schmidt-Heydt

**Affiliations:** 1 Department of Safety and Quality of Fruit and Vegetables, Max Rubner-Institut (MRI), Federal Research Institute of Nutrition and Food, Karlsruhe, Germany; Vanderbilt University, Nashville, Tennessee, USA

**Keywords:** aflatoxin, *Aspergillus parasiticus*, high-quality genome sequence

## Abstract

*Aspergillus parasiticus* is an important aflatoxigenic fungus, frequently found in soil samples. Here, we report the sequencing of *A. parasiticus* strain MRI410 using Illumina MiSeq and Oxford Nanopore platforms. This strain was isolated from soil of a Kenyan maize field.

## ANNOUNCEMENT

The filamentous fungus *Aspergillus parasiticus* is, in addition to *A. flavus*, the most serious aflatoxigenic *Aspergillus* section *Flavi* species. It is described to prefer ground crop hosts to plant hosts and was already repeatedly isolated from peanuts (1, 2). To our knowledge, genomes of *A. parasiticus* have only been sequenced by short-read sequencing technologies until now. To provide a genome assembly of an *A. parasiticus* strain sequenced also with a long-read sequencing technology, strain MRI410 was sequenced using Oxford Nanopore in addition to Illumina MiSeq technology.

**Fig 1:**
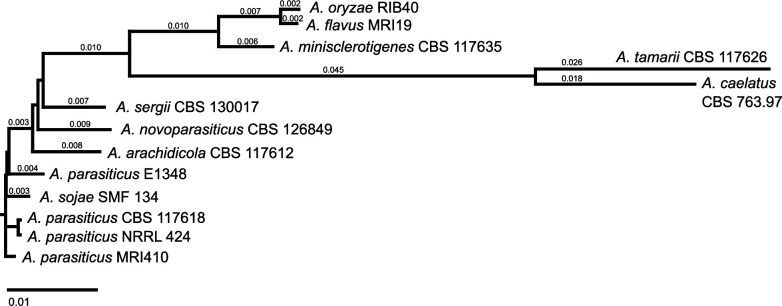
Phylogenetic tree of different *Aspergillus* species. *A. parasiticus* MRI410 was initially identified by sequencing PCR products of partial β-tubulin [Bt2a/2b (3)], calmodulin [cmd5/6 (4), cmd2F/2R (5)], and nitrate reductase [niaDF/AR (5), niaDBF/BR (5, 6), niaDCF/CR (7)] genes. The recommended mastermix composition of the peqGOLD Taq DNA polymerase all-inclusive kit (VWR International GmbH, Darmstadt, Germany) with 2.5 µL of each primer (5 pmol/µL) and 5 µL DNA was used for the PCR. The following cycling program was chosen: 95°C for 3 min; 40 cycles of 95°C for 30 s, 52°C (cmd2F/2R)/55°C (niaDF/AR)/57°C (niaDBF/BR, niaDCF/CR)/60°C (Bt2a/2b, cmd5/6) for 40 s, and 72°C for 90 s; and 72°C for 3 min. PCR products were Sanger sequenced by Eurofins Genomics (Cologne, Germany). Forward and reverse sequences were assembled with SeqMan Pro (LaserGene v17, DNASTAR, Inc, Madison, WI, USA). For calmodulin and nitrate reductase, the two, respectively, three overlapping consensus sequences were concatenated using MegAlign Pro and SeqBuilder Pro (LaserGene v17, DNASTAR, Inc, Madison, WI, USA). Resulting sequences were compared to the database of the NCBI using BLASTN. To generate a phylogenetic tree using the neighbor-joining algorithm, the three partial genes were concatenated and aligned (Mauve, MegAlign Pro, LaserGene v17, DNASTAR, Inc, Madison, WI, USA) with the same sequences of other *Aspergillus* species [*A. arachidicola* CBS 117612: ML737115.1, ML737234.1, ML737155.1 (8); *A. caelatus* CBS 763.97: NW_022475357.1, NW_022475408.1, NW_022475603.1 (8); *A. flavus* MRI19: JAGYXF010000057.1, JAGYXF010000047.1, JAGYXF010000013.1 (9); *A. minisclerotigenes* CBS 117635: ML732812.1, ML732765.1, ML732764.1 (8); *A. novoparasiticus* CBS 126849: ML733430.1, ML733443.1, ML733467.1 (8); *A. oryzae* RIB40: NC_036440.1, NC_036436.1, NC_036438.1 (10); *A. parasiticus* NRRL 424: MK119739.1, MK119705.1, MK119671.1 (6); *A. parasiticus* CBS 117618: ML734942.1, ML734938.1, ML734939.1 (8); *A. parasiticus* E1348: SJFE01000042.1, SJFE01000024.1, SJFE01000049.1 (11); *A. sergii* CBS 130017: ML741807.1, ML741799.1, ML741762.1 (8); *A. sojae* SMF 134: CP035525.1, CP035529.1, CP035527.1 (12), *A. tamarii* CBS 117626: ML738700.1, ML738591.1, ML738590.1 (8)].

For the isolation of this strain, soil samples from the rhizosphere of a maize field in Makueni (1.382 S, 37.322 E), Kenya, were diluted using Tween-80/NaCl-mixture (NaCl 9 g/L, Tween-80 1 g/L, and agar 1 g/L), and serial dilutions were incubated on selective nutrient media. Then, individual colonies were separated. The strain was identified by partial sequencing of β-tubulin, calmodulin, and nitrate reductase genes (Fig. 1), and the identification was verified by the Westerdijk Fungal Biodiversity Institute (Utrecht, Netherlands). After the incubation of *A. parasiticus* MRI410 on malt extract agar (malt extract 17 g/L, glucose 5 g/L, and agar 16 g/L) at 25°C for 4 d and its homogenization in liquid nitrogen, DNA was isolated using the NucleoSpin Plant II kit (Macherey-Nagel, Düren, Germany) following the official protocol. DNA quality and concentration were verified using a NanoDrop 1000 spectrophotometer and a Qubit 3.0 fluorometer (both Thermo Fisher Scientific GmbH, Bremen, Germany). The same DNA sample was used for the following sequencing with Oxford Nanopore and Illumina MiSeq technologies.

For long-read sequencing, a library was prepared using the Rapid Sequencing kit (Oxford Nanopore Technologies, Oxford, United Kingdom). Sequencing was performed on a MinION Mk1C instrument (Oxford Nanopore Technologies, Oxford, United Kingdom) resulting in 573,070 raw reads (1.56 Gb, N_50_ 6.35 kb). Subsequent base calling of Nanopore data was done using Guppy, and quality was checked using pycoQC (13). For short-read sequencing, a library was generated using the DNA Prep kit (Illumina, San Diego, CA, USA), and its quality was controlled with the Experion DNA 1k analysis kit (Bio-Rad, Feldkirchen, Germany). The 2 × 300 bp sequencing was performed on a MiSeq platform (Illumina, San Diego, CA, USA). A total of 26,943,603 raw read pairs were trimmed (Trimmomatic-0.39) based on the quality control (FastQC 0.11.3) (14).

Default software parameters were used except where otherwise noted. A d*e novo* assembly of the Nanopore data was generated using Flye 2.8.2 (average coverage: 22.9×) (15, 16). Subsequently, the MiSeq data were aligned (average coverage: 235.2×) to the resulting contigs using BWA 0.7.17, SAMtools 1.10, and QualiMap 2.2.1 (17 - 19), and the alignment was polished with Pilon 1.23 and SAMtools 1.10 (without activating the IUPAC nucleotide codes) (16, 20). The resulting assembly consisted of 60 contigs and a total of 38.68 Mb (N_50_: 2,119,486 bp, GC content: 47.68%) of gDNA. A further contig contained the complete mitochondrial DNA (29,136 bp). To assess the completeness of the genome assembly, a BUSCO (v5.4.6) analysis using the lineage database ascomycota_odb10 was performed (21). A total of 1,638 (96.0%) complete single-copy and 15 (0.9%) complete duplicated orthologs, 2 (0.1%) fragmented orthologs, and 51 (3.0%) missing orthologs were retrieved.

In total, 55 gene clusters coding secondary metabolites were predicted using antiSMASH 6.1.0 with the cluster finder algorithm for BGC border prediction (22, 23). The aflatoxin gene cluster will be further analyzed and compared to that of other *Aspergillus* species.

## Data Availability

This Whole Genome Shotgun project has been deposited at DDBJ/ENA/GenBank under the accession no. JAMRJN000000000 and BioProject accession no. PRJNA835319. The version described in this paper is version JAMRJN010000000. The raw sequence reads have been deposited in the Sequence Read Archive (SRA) under accession no. SRX17682209 and SRX17682210. The partial gene sequences have been deposited at the GenBank under accession no. OQ909814, OQ909817, and OQ909820.
